# Genomic-based identification of environmental and clinical *Listeria monocytogenes* strains associated with an abortion outbreak in beef heifers

**DOI:** 10.1186/s12917-020-2276-z

**Published:** 2020-02-22

**Authors:** Katherine J. Whitman, James L. Bono, Michael L. Clawson, John D. Loy, Joseph M. Bosilevac, Terrance M. Arthur, Jeff D. Ondrak

**Affiliations:** 1 University of Nebraska-Lincoln, School of Veterinary Medicine and Biomedical Sciences, Great Plains Veterinary Educational Center, Clay Center, NE 68933 USA; 2 USDA ARS US Meat Animal Research Center, Clay Center, NE 68933 USA; 3 University of Nebraska-Lincoln, School of Veterinary Medicine and Biomedical Sciences, Nebraska Veterinary Diagnostic Center, Lincoln, NE 68583-0907 USA

**Keywords:** Cattle abortion, Listeriosis, Silage, Whole-genome sequencing, Outbreak

## Abstract

**Background:**

In a beef cattle facility an outbreak of abortions occurred over a 36-day period and included samples from two aborted (non-viable) fetuses and 21 post-abortion clinical cases. There are numerous etiologies, including clinical listeriosis. At the species level, *Listeria monocytogenes* is ubiquitous in cattle production environments, including soil, feed, and occasionally water sources, and is a common enteric resident of cattle and other mammals. There are four genetically distinct lineages of *L. monocytogenes* (I-IV), with most lineage III and IV isolates obtained from ruminants. Definitive diagnosis of *L. monocytogenes* as a causative agent in disease outbreaks relies upon case identification, appropriate sample collection, and laboratory confirmation. Furthermore, clearly establishing a relationship between a pathogen source and clinical disease is difficult.

**Results:**

Of the two fetal and 21 clinical case submissions, 19 were positive for *L. monocytogenes*. Subsequent culture for *L. monocytogenes* from water and silage sources identified both as potential origins of infection. Using whole-genome sequencing and phylogenetic analyses, clinical, water and silage *L. monocytogenes* strains grouped into two of four lineages. All water and silage strains, plus 11 clinical strains placed in lineage III, with identical or nearly identical genomic sequences. The remaining eight clinical strains placed in lineage I, with seven having nearly identical sequences and one distinctly different.

**Conclusion:**

Three genetically distinct strains within two lineages of *L. monocytogenes* caused the abortion outbreak. The etiology of abortion in 11 cases was directly linked to water and silage contamination from a lineage III *L. monocytogenes* strain. The source of infection for the remaining abortion cases with two different strains from lineage I is unknown. This is the first report of *L. monocytogenes* genomics being used as part of an outbreak investigation of cattle abortion.

## Background

*Listeria monocytogenes* is a well-known saprophytic bacterial pathogen that is ubiquitous in the cattle production environment. Cattle and many other mammals including humans, can be asymptomatic shedders. However, *L. monocytogenes* can cause a number of diseases across species, including visceral listeriosis, neurologic listeriosis, and importantly, reproductive listeriosis, which is implicated in late-term abortion in cattle. Certain populations of cattle, particularly those who are immunocompromised, pregnant, young, old, or stressed are more affected by environmental risk factors associated with *L. monocytogenes* infection [1–3].

The potential routes of infection leading to clinical listeriosis in cattle is either ingestion, inhalation, direct contact or oral mucosal lesions [4–7]. The primary route in cattle is suspected to be the consumption of contaminated feed or water and subsequent passage through the gastrointestinal tract, a pathway that has been demonstrated in goats and sheep [8, 9]. Once in the gastrointestinal tract, *L. monocytogenes* is able to bind, penetrate and move within and between the epithelial cells [10]. Translocation of *L. monocytogenes* occurs from the intestines to the liver and hepatocytes via macrophages, leading to a bacteremia that results in either an effective cell-mediated immune response, neurological disease, or reproductive infection [11].

Across mammalian species, the incubation period from infection to clinical signs of listeriosis is variable, generally 2–6 weeks [12]. Consequently, the delayed onset of clinical signs following infection makes identification of a potentially contaminated feed source challenging, in that the feed may have been entirely consumed by the time clinical listeriosis is observed. Even if *L. monocytogenes* is detected in available feed, the many available molecular diagnostic techniques [13] may not be specific enough to directly link the source strain of *L. monocytogenes* to the clinical strain due to its pervasiveness in the environment.

*L. monocytogenes* diversity has been assessed using various methods. Historically, serotyping [14] and pulsed-field gel electrophoresis (PFGE) [15, 16] have been implemented in source origin identification of strains in human and cattle listeriosis outbreaks. However, these diagnostics do not definitively establish genetic relationships between strains. Genomic sequencing has advantages over traditional diagnostics in that it interrogates the complete genome and can establish a more definitive relationship between isolated strains. In situations where *L. monocytogenes* can be established as an etiology for clinical disease as well as an environmental contaminant, utilizing genomic sequencing can verify that a specific strain found in the environment is responsible for clinical disease.

There are four genetically distinct lineages of *L. monocytogenes* (I-IV) that have been found across mammalian species and in the environment [17, 18]. *L. monocytogenes* strains can be classified into one of four lineages based on a number of techniques including: pulse-field gel electrophoresis, ribotyping [14, 19], multi-locus enzyme electrophoresis, multilocus sequencing typing and more recently whole genome sequencing. Of the four lineages, strains from lineage III are often isolated from cattle, and lineage IV is currently exclusive to ruminants. Lineages I through III have been documented in clinical listeriosis in cattle and other mammals [17].

In this report, an outbreak investigation of *L. monocytogenes* abortion in a beef cattle operation is described. Aborted bovine fetuses, cervico-vaginal (CV) swabs, and retained fetal membranes (RFM), if present, from aborting females, as well as their water and feed sources were sampled and cultured for *L. monocytogenes*. To establish a definitive source of the abortion outbreak, all *L. monocytogenes* culture positive strains were subjected to whole-genome sequencing, phylogenetic analyses, and lineage classification.

## Results

### Cattle

**Fig. 1:**
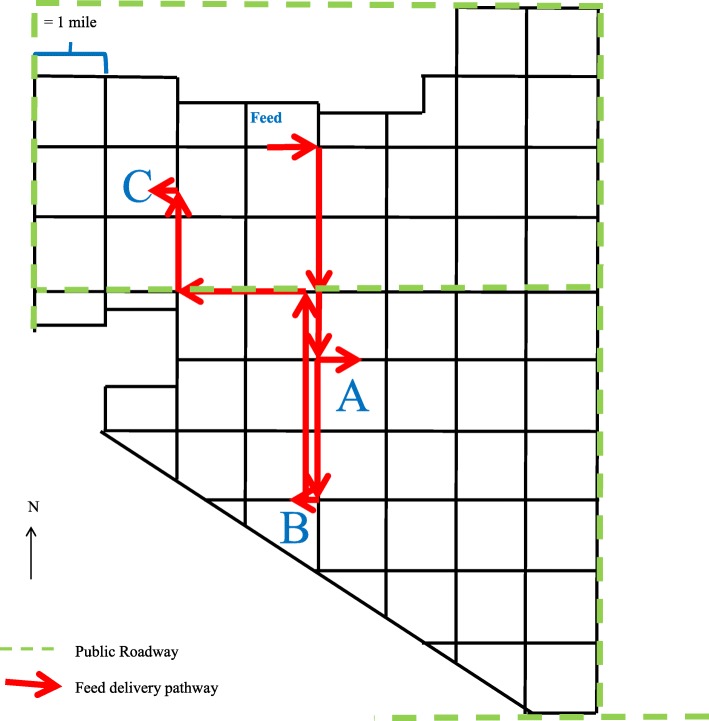
Layout of operation and feed pathway from shared feed location

An abortion outbreak occurred at the United States Meat Animal Research Center (USMARC) in late winter/early spring of 2014. A total of 28 heifers aborted over a 36-day period, with 24 abortions occurring in a 12-day period. By comparison, four mature cows aborted during the calving season, none within the acute abortion outbreak timespan and none were positive for *L. monocytogenes*. Three heifer management sites (A-C) were affected, and all were managed similarly, fed from the same silage source, and from the same feed truck (Fig. 1). Two- and three-year old females were never grouped with mature cows, but rather rotated through pastures, occupying sites where cows had resided approximately 7 months previous. Females were aborting late term (3rd trimester) fetuses, with no other observed clinical signs, except some with blood on the perineum or RFM, and some groups had refused feed as recently as the day prior to the first abortion. Descriptive statistics of abortions during the calving season are listed in Table 1. The mature cow known abortion percentage was 0.1%, but was at 3% for the heifer population, much higher than spring calving herd reports by the National Animal Health Monitoring System (NAHMS) [20]. The majority of abortions occurred within a 12-day time period, prompting the outbreak investigation.

**Table 1:** Descriptive statistics relative to the female population over the calving season

Category	Total Population	Season Abortion Count	Season Abortion %	Day 1–12 Abortion Count	% Total Abortions Occurring Day 1–12
Mature Cows	3633	4	0.1%	0	0
Heifers	936	28	3.0%	24	89%
Total	4569	31	0.7%	24	77%
Site A	369	17	4.6%	16	94%
Site B	283	3	1.1%	3	100%
Site C	284	8	2.8%	5	71%

During the outbreak, two aborted bovine fetuses were submitted to the University of Nebraska Veterinary Diagnostic Center, Lincoln, NE (UNLVDC), for aerobic culture, *C. fetus* culture, *Leptospira spp.*, Bovine herpesvirus 1 (BHV1), bovine viral diarrhea virus (BVDV) PCR, and histopathology. Cervico-vaginal swabs or RFM from 21 of the 28 aborting heifers were also collected on follow-up sampling and submitted to the UNLVDC for aerobic culture, followed by *L. monocytogenes* culture and/or *L. monocytogenes* PCR, if needed. Both fetuses and 17/21 heifer samples were positive for *L. monocytogenes,* specifically 11/11 RFM and 6/10 CV swabs. Fetal tissues were examined by board certified pathologists as part of the abortion diagnostic investigation. Fetus 1 had liver lesions consistent with listeriosis and included scattered foci of acute necrosis with moderate neutrophilic inflammatory response. Fetus 2 had marked autolysis within the liver with presence of few mononuclear cells in the hepatic capsule. Both pathologists diagnosed listeria as the cause of abortion. Fetuses were negative to all other diagnostics, including PCR for *Leptospira* spp., BHV1 and BVDV, and culture for *Campylobacter* sp. No other diagnostics were performed on the RFM or CV samples. Descriptive statistics of the sample submissions and results are summarized in Table 2.

**Table 2:** Descriptive statistics of clinical submissions

Sample ID	Sample Type	Site Location	Aerobic Culture	LM PCR	LM Culture
1	F	A	+		
2	F	B	+		
3	S	A	–	–	–
4	P	A	+		
5	S	A	+		
6	S	A	–	–	–
7	S	A	+		
8	S	A	+		
9	P	A	–	+	
10	S	A	–	–	–
11	P	C	+		
12	P	C	+		
13	P	A	+		
14	P	A	+		
15	P	A	+		
16	P	A	+		
17	P	A	+		
18	S	A	–	–	–
19	S	C	–	+	
20	S	A	+		
21	S	C	+		
22	P	C	+		
23	P	A	+		

*LM L. monocytogenes*, *F* Fetal tissues, *P* Retained fetal membranes, *S* Cervico-vaginal (CV) swabs

### Feed and water samples

Nine water sources were tested, and four were culture positive for *L. monocytogenes* (Table 3). Site A had two water tanks positive for *L. monocytogenes* while site B contained one positive water tank and one positive water hole by the feed bunk. Water tanks associated with site C were sampled, but no *L. monocytogenes* was isolated. Of the 15 silage samples tested, *Listeria* spp. were detected in 14 samples, of which four samples were culture positive for *L. monocytogenes*. Of those positive for *L. monocytogenes*, three strains were derived from feeds that had a pH less than 5.0. Feed sample timing, type, location, pH, and test results are summarized in Table 4. Sample locations of the pile face correspond to Additional file 1.

**Table 3:** Summary of *L. monocytogenes* water samples

Sample	Site	Direct LM PCR	Enriched LM PCR	Enriched LM Culture
Tank 1	A	**+**	**+**	**+**
Tank 2	**A**	**–**	**+**	**+**
Tank 3	**B**	**–**	**+**	**+**
Tank 4	C	**–**	**–**	**–**
Hole 5^a^	**A**	**–**	**+**	**–**
Hole 6	B	**–**	**+**	**+**
Hole 7	B	**–**	**–**	**–**
Hole 8	C	**–**	**–**	**–**
Hole 9	C	**–**	**–**	**–**

^a^Culture positive for *L. innocua*-not sequenced

**Table 4:** Summary of *L. monocytogenes* positive feed samples

Sample timing	Pile location	pH	LM culture	LM PCR
1st	1	4.54	+	+
1st	10	8.09	+	+
2nd	1	7.09	+	+
2nd	5	4.34	+	+

Sample timing: 2nd sample collected 10 days after 1st sample. All positive samples were from corn silage. Pile location is in reference to the position on the silage face (Additional file 1)

### Genomics

Thirty one strains were subjected to short read Illumina sequencing in this study, of which 27 were part of the USMARC outbreak and four were available from the UNLVDC (see Additional file 2 for strain information). All 31 strains passed post-sequencing processing for low quality reads and adapter sequence. The lowest read coverage was 12.8X while the highest coverage was 132X with the average being 58X. Parsnp was used to create an initial phylogenetic tree based on Strategic Kmer Extension for Scrupulous Assemblies (SKESA) assembled chromosomes of the 31 sequenced strains. The tree was divided into three major lineages with six minor clusters. One strain from each cluster of the initial tree was also sequenced using long-read PacBio sequencing to generate reference complete closed genomes for each cluster.

The four major *L. monocytogenes* lineages were reproduced in a maximum-likelihood tree calibrated with 20 *L. monocytogenes* chromosomes of known lineage and serotype affiliations that were available from the National Center for Biotechnology Information (NCBI, Additional file 2: Fig. 2). The tree also contained 25 SKESA assembled *L. monocytogenes* chromosomes and six closed PacBio generated chromosomes from this project that were assigned to lineages based on their placement in the tree. Lineage I contained seven NCBI genomes representing serotypes 1/2b, 3b, 4b, 4d, 4e, and 7, and eight clinical strains from cattle located in two different USMARC management sites (A and C) and one UNLVDC strain. Seven of the lineage I clinical *L. monocytogenes* strains molecularly serotyped as 1/2b based on their in silico multi-locus sequence typing (MLST) patterns, and one molecularly serotyped as 4b.

**Fig. 2:**
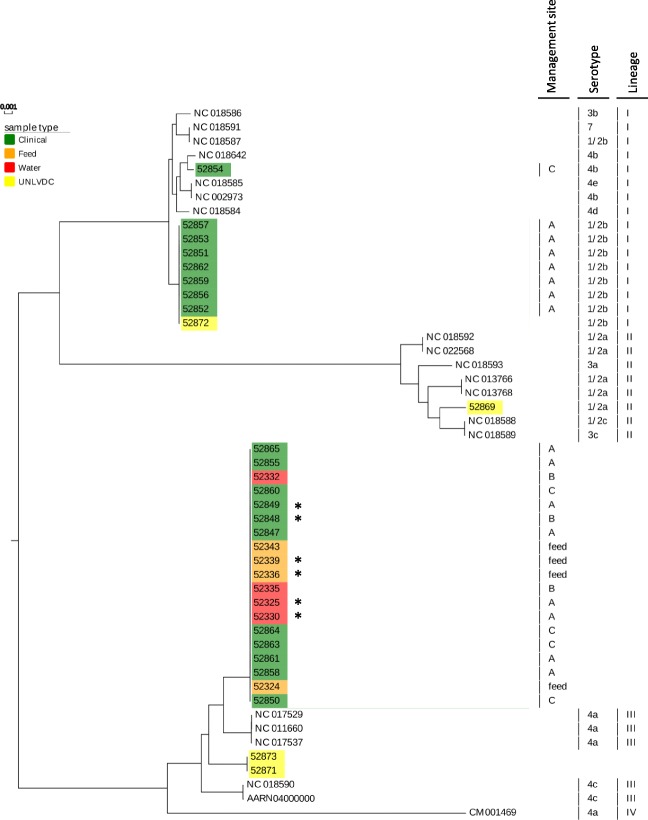
Parsnp generated tree of complete closed or draft genomes of *L. monocytogenes*

Lineage II contained seven genomes from NCBI that represented serotypes 1/2a, 1/2c, 3a and 3c plus one UNLVDC strain that molecularly serotyped as 1/2a (Additional file 2: Fig. 2). No lineage II strains were isolated from any of the three cattle management sites, feed, or water. Lineage III contained two NCBI genomes that represented serotypes 4a and 4c, and two UNLVDC strains that could not be molecularly serotyped because there were no matching MLST allele patterns in the Listeria Pasteur MLST or Center for Genomic Epidemiology databases [21, 22]. Lineage III also contained a monophyletic cluster of eight environmental strains and eleven clinical strains that collectively originated from feed and water or cattle at all three sites, respectively (Additional file 2: Fig. 2). These strains also could not be molecularly serotyped based on their MLST allele patterns.

While all clinical strains from the USMARC abortion outbreak placed in either lineages I or III, the feed and water strains only placed in lineage III. Of the eight USMARC clinical strains that placed in lineage I seven grouped with a UNLVDC strain and were either identical or differed from each other by no more than six single nucleotide polymorphisms (SNPs). In contrast, the UNLVDC strain differed from the seven clinical strains by more than 60 SNPs. These seven clinical strains were all isolated from cattle at site A and molecularly serotyped as 1/2b. The other clinical strain in lineage I was isolated from a heifer at site C and was more closely related to NCBI genome NC_018642, a 4b serotype isolated from cheese [23]. Consequently, lineage I contained two genetically distinct *L. monocytogenes* strains that originated from clinical cases in two different management sites with no apparent connection to strains isolated from feed and water samples.

All 11 clinical strains that placed in lineage III were either identical or no more than eight SNPs different from the lineage III strains recovered from the silage pile. Additionally, two clinical, two water and two corn silage strains were identical across their core genome, which represented 99% of a closed, circular, PacBio-generated reference genome for isolate 52,330 (Additional file 3). Consequently, all lineage III strains from the outbreak were either completely clonal, or only differed by a few SNP alleles. These results indicate that there were three genetically distinct *L. monocytogenes* strains responsible for the cattle abortion outbreak, with two that placed in lineage I and one that placed in lineage III.

## Discussion

Although an important diagnostic sample, only two aborted fetuses were recovered by cattle managers for *L. monocytogenes* testing in this outbreak. Consequently, alternative sampling was needed to confirm the etiology of the abortions and to quickly and efficiently implement appropriate management changes. To that end, CV swabs or RFM from females that aborted proved to be valuable diagnostic samples in this outbreak when fetuses were unavailable. An etiologic diagnosis was achieved in 81% (17/21) of all examined clinical cases, with 100% (11/11) of the retained placental samples and 60% (6/10) of the CV swabs positive for *L. monocytogenes* (Refer to Table 2). This high rate of diagnostic success was achieved despite sampling being delayed up to 7 days from the time of abortion. Additionally, 17/19 of the overall positive cultures (including two fetal tissue submissions) were isolated from primary aerobic culture and did not require additional enrichment. This is important as cold enrichment of *L. monocytogenes* can take weeks, further delaying the time to a diagnosis.

Diagnostic investigation indicated the causative agent for the abortion outbreak was *L. monocytogenes*, however the source of the organism(s) was not immediately clear. A positive *L. monocytogenes* result from a day one fetal tissue sample submission was not reported until day eight of the outbreak, with 19 abortions occurring within that week. Mitigating the abortion outbreak was critical for the remaining pregnant females in the herd. Previous literature indicated that poorly ensiled feed was associated with *L. monocytogenes* contamination [24–28]. Because of this, focus was placed on the supplemental silage as a potential source, and feeding was discontinued on day eight until results from the remaining case samples and environmental testing were completed. Abortions cases ceased 4 days after removing suspected silage from the ration, with the exception of one occurring 19 days later. The reason for the abrupt halt to the abortions following silage removal is unclear. The incubation period for *L. monocytogenes* would suggest that abortions should have continued for a longer time if exposure occurred and the dose was sufficient. Immediately after day one fetal tissue sample submission results were confirmed positive for *L. monocytogenes*, a novel and organized approach to feed and water sampling was employed, resulting in more effective and efficient collection of environmental samples and subsequent source identification.

Both silage and water samples were culture positive for *L. monocytogenes*. Of the four positive water samples, three required enrichment to facilitate growth, indicating a smaller bacterial load in these sources. Therefore, we suspected early on in the investigation that the source of *L. monocytogenes* in the water was likely from heifers transferring feed into the water soon after consuming affected silage. Interestingly, some feed samples positive for *L. monocytogenes* were at a pH of less than 5.0, which should have been lethal to the bacteria. This finding is supportive of other research indicating that *L. monocytogenes* can survive at a pH of less than 5.0 [26, 29], bringing the traditional acceptable pH for appropriately ensiled feeds into question.

While clinical and laboratory findings were supportive of *L. monocytogenes* in the environment as the likely etiology for the abortions, we wanted to determine if a genetic relationship between the isolates from the environment and clinical samples existed and if the feed was the source of *L. monocytogenes*. Disposing of several hundred tons of silage had significant economic and resource implications, but herd health and welfare related to feeding contaminated silage was a major concern, and the cattle could be exposed to other risk factors associated with *L. monocytogenes* infection, such as oral-fecal transmission and the ubiquitous nature of the bacteria. Proving that the silage was the source of the outbreak, and not another cause would further strengthen the decision to dispose of all contaminated silage.

To our knowledge, full genomic sequencing of *L. monocytogenes* has not been previously used in a bovine abortion outbreak investigation. Using this technique would give the highest resolution for determining the source of the outbreak. Two silage, two water, and two clinical *L. monocytogenes* strains were identical to each other, and nearly identical to all remaining silage (*n* = 2) and water (n = 2) strains, and clinical strains (*n* = 9). Thus, the same lineage III genetic strain type was found in silage, water, and clinical case samples. The direction of the feed delivery pathway (Fig. 1) would not have allowed for water or cattle to contact the original silage pile. This implicated the silage as the most likely source of the pathogen and the cause of abortion in at least 11 heifers.

The remaining eight *L. monocytogenes* positive clinical strains consisted of two genetically distinct lineage I strain types. Females infected with either of the two lineage I strain types were kept with other heifers that were infected with the distinct lineage III strain type that traced back to the silage. Of note, when silage feeding was discontinued, all abortions but one ceased 4 days later. Thus, it is possible that all three genetically distinct strains implicated in the outbreak could have been in the feed, with two undetected. Importantly, identification of seven lineage I clinical *L. monocytogenes* strains as sertotype 1/2b provided risk information for cattle handlers. Serotypes 1/2b and 4b, along with serotype 1/2a, are the main serotypes that cause of human disease and represents 90–95% of cases [30–32]. Consequently, personnel working with cattle from the outbreak were at an increased risk to become infected with *L. monocytogenes*.

Because *L. monocytogenes* was isolated from the silage at different sampling times and at different depths, the integrity of the entire silage pile was called into question, and therefore it was decided that the feed was unsuitable for further use. The remaining affected silage pile was spread onto pastures that would not be populated for several months, so that UV light and desiccation could synergistically eradicate the remaining *L. monocytogenes* [33–35]. In addition to routine cleaning of the water tanks, recommendations for future silage management were made including: appropriate packing of newly harvested silage to ensure an anaerobic environment within the pile, vermin mitigation, surface protection and routine silage pile sampling throughout the subsequent feeding season to monitor pH and *L. monocytogenes* presence. The silage sampling scheme would follow the protocol used in the investigation, with suggested 2–4 week sampling intervals.

Management strategies to prevent another abortion outbreak due to *L. monocytogenes* appeared to be effective in subsequent production years. Following the acute outbreak, all additional aborted fetal submissions screened for *L. monocytogenes* in the 2014 calving season and following seasons did not detect or isolate *L. monocytogenes*. Additionally, no clinical listeriosis cases of any kind in the cowherd were observed during that time. No negative sequelae to reproductive performance was observed in the recovered animals. Of the 17 *L. monocytogenes* positive females, five were culled prior to the 2014 breeding season, and the remaining 12 were diagnosed pregnant (100%) by ultrasound in the fall of the same year. In the 2015 calving season, those heifers all carried a term calf. Eleven of 12 calves were born alive, with dystocia as the documented cause of the only calf death loss, resulting in a 91.6% calving percentage for those females exposed.

## Conclusion

This case investigation resulted in a very complete epidemiologic picture of an *L. monocytogenes* abortion outbreak in beef cattle. *L. monocytogenes* was quickly identified as the cause through strategic sampling of affected cattle and their environment, with contaminated silage initially implicated as the probable source. The outbreak was brought under control through the elimination of the contaminated silage. Subsequent whole genome sequencing showed that three strains were involved in the outbreak and confirmed that silage was the primary source of at least one of them.

Abortions in beef cattle can be a major problem in herds, particularly if a larger than normal percentage of the population is affected, or if outbreaks occur over a short period of time. During *L. monocytogenes* abortion outbreaks, challenges in timely disease recognition and diagnosis can create limitations for treatment of affected cattle and management decisions. Thus, quickly establishing a clear connection between the host, pathogen, and environment through strategic animal and environmental sampling, followed by strain identification using whole genome sequencing of *L. monocytogenes* isolates allows for appropriate management of feed and environmental risks, as well as strategies for risk management of future outbreaks. Using whole-genome sequencing of pathogens in outbreak investigations will give veterinarians and epidemiologists greater flexibility and stronger evidence for confirming the strains and sources involved.

## Methods

### Cattle population and management

All sampled cattle in this report resided at the USMARC in Clay Center, Nebraska, and sampling protocols were approved by the Institutional Animal Care and Use Committee at the University of Nebraska, Lincoln (IACUC, #1383). Veterinary intervention and sampling were requested by the owner soon after the abortions began. The entire cattle population was considered closed; bovine semen was the only source of new genetic material and biosecurity measures were in place to prevent direct contact with outside cattle. Staff and equipment were shared between cattle locations within USMARC.

As a part of routine management practices, all females were previously vaccinated for *Brucella abortus* and given an initial *Campylobacter fetus* and *Leptospira canicola-grippotyphosa-hardjo-icterohaemorrhagiae-pomona* bacterin (Spirovac VL5, Zoetis, Kalamazoo, MI), followed by a modified live IBR, BVD types 1 and 2, and PI_3_ vaccine in combination with *C. fetus*, and *Leptospira spp.* bacterin (PregGuard Gold FP 10, Zoetis, Kalamazoo, MI) 30 days before the 2013 breeding season. Pregnancy was diagnosed via rectal ultrasound between 45 and 100 days gestation. A total of 28 females (27 two-year old heifers and 1 three-year old female, herein referred to as “heifers”) aborted in late winter/early spring of 2014. Twenty-one of these cases were sampled (described below) and diagnostics performed at the UNLVDC in Lincoln, Nebraska.

All pregnant females were on pasture prior to, during, and after all sampling. During the winter months, all cattle were supplemented primarily with corn silage stored and fed from the same feed storage site. Silage was analyzed for nutritional content and combined with mineral supplementation to meet nutritional requirements. Rations were delivered by the same truck and dispensed in mobile bunk or tire feeders. These feeders were moved periodically when conditions around the bunks were deemed unsanitary. Heifers that aborted were from one of three management sites (A-C) that were managed in a similar fashion. A site map of the operation with feed delivery pathway is identified in Fig. 1.

### Clinical case sampling

Twenty-eight heifers and four mature cows (4 years or greater) aborted by the end of the 2014 calving season, with 24 heifer abortions occurring over 12 days. All collected tissues and sampling of females that had aborted were performed by or under the direct supervision of a licensed veterinarian (lead author). Aborted fetal tissues, including fresh and fixed heart, lung, liver, spleen and kidney, as well as stomach contents and ear notches, were submitted to the UNLVDC on day one and day eight of the investigation, the days that they were discovered and recovered by cattle managers. All fetal tissues were placed in insulated cooler with icepacks and shipped overnight to the UNLVDC.

Of the 28 heifers that were identified by cattle managers and suspected of aborting, 21 were humanely restrained in a commercial cattle chute and were subjected to individual examination and sample collection (day 8, 14, or 27). The perineum of each female was cleaned with soap and water, rinsed and dried, and the tail held away from the region. If RFM were present, a clean, gloved hand was inserted into the vagina and the membranes were extracted caudally until the hand and membranes were outside the vulva, then a section of membrane was aseptically removed and placed in a sterile collection bag. In heifers that did not have RFM, a uterine culture double-guarded swab (Jorgensen Laboratories, Loveland, CO) was used to sample near and within the cervical opening (CV swab). To facilitate the entry of the swab, a disposable lubricated vaginal speculum was used to visualize the cervical opening and the area just proximal to the opening. Approximately 1–2 cm of the distal cervix and the area just proximal to the opening were swabbed. This method prevented undue contamination from the vagina, which in many cases contained discharge and fluid as a result of metritis or pyometra. Once swabs were collected, samples were contained within the protective unit of the swab mechanism. All case samples were placed in insulated cooler with icepacks and shipped overnight to the UNLVDC. Heifers were released after sampling into communal pens for health observation, then subsequently released back onto pasture.

### Feed and water sampling for *L. monocytogenes* and pH

Supplemental feed consisted of corn silage, earlage, and haylage separated into open concrete bunkers. Corn silage and earlage were sampled by visually dividing the piles into three stratified layers (top, middle and base) and crosswise sections (left, middle, and right) to provide targeted and documented sampling locations (Additional file 1). Samples were obtained by first brushing away loose materials on the surface that were potentially transferred or contaminated by feeding equipment. Exposed feed was then collected at a depth of approximately 10 cm. Additional samples included loose surface material from the center face and apron of the piles as well as loose material present in the drainage tube of each pile. Samples were collected from haylage in a similar fashion as silage piles but from only three of the quadrants: center-top, mid-left and bottom-right. All samples were collected with freshly gloved hands, placed in sterile bags, and transported to the laboratory where they were held at 4 °C until processing. Silage and earlage pile samples were collected a second time, 10 days after the initial sampling, and were processed in the same manner. The second sample was taken after removal of at least one meter of the silage face to determine if contamination existed deeper within the silage piles, or if contamination was only present in isolated areas. Thirty-six feed samples were collected over this period.

Accessible water either from a tank or standing water in close proximity to the tank was collected where clinical cases of listeriosis had been identified. Nine water samples (three from each management site) were obtained by placing a 50 mL screw cap conical test tube into the water source. A scooping motion was used, such that the bottom of the tank or water hole was contacted by the tube to capture any sediment present. Tubes were capped and transported immediately to the laboratory for processing. Water sources were only sampled once. After sampling, water tanks were drained, disinfected, allowed to dry, and refilled.

To measure the pH of the feed, collected samples were placed in a 100 mm petri dish then wetted with approximately 10 mL sterile distilled deionized water. A surface pH meter (ExTech Instrument Corp., Nashua, NH) was used to measure the pH of the moistened surface. For each dish of silage, average pH was determined by measuring pH at three separate areas corresponding to 12, 4 and 8 o’clock positions in the petri dish.

### *L. monocytogenes* detection and isolation from clinical samples

All diagnostic specimens collected during the case investigation were shipped overnight in insulated coolers with icepacks to the UNLVDC. Submitted samples included fresh and fixed fetal tissues (collected and submitted on day 1 and 8) and CV swabs or RFM from the 21 sampled heifers (day 8, 14, and 27). Requested diagnostics for fetal tissues included: aerobic culture and sensitivity, *C. fetus* culture, *Leptospira spp.*, BHV1, and BVDV PCR, and histopathology. Fetuses and fetal tissues, including spleen, heart, lung, kidney, and liver were fixed in 10% neutral buffered formalin, embedded in paraffin, sectioned and stained with hematoxylin and eosin, examined histopathologically by board certified pathologists with other ancillary testing at their discretion to determine a diagnosis. Testing at UNLVDC is by standard operating procedures accredited under the American Association of Veterinary Laboratory Diagnosticians (AAVLD). Fetal tissues (lung and liver) and stomach contents were subjected to culture testing. Requested diagnostics on the CV and RFM samples collected included aerobic culture and bacterial identification.

Fetal tissue samples were flame sterilized and directly plated on to TSA with 5% sheep’s blood, chocolate agar, Colombia CNA agar with naldixic acid and 5% sheep’s blood (CNA), Campy CVA agar and MacConkey’s agar (Thermo Fisher Scientific, Waltham, MA). Cervico-vaginal swabs, RFM, and fetal stomach contents were directly plated onto the same agar media as the tissues. Remaining samples were macerated (tissues) or agitated (swabs) into Fraser’s Broth (Thermo Fisher Scientific, Waltham, MA). All media except Campy CVA were incubated for 18–24 h at 37 °C with 5% CO_2_ supplementation, then examined by trained laboratory technicians following the UNLVDC Standard Operating Procedures. Media without pathogenic bacterial growth were re-incubated and observed following an additional 18–24 h incubation. Campy CVA agar was incubated 48 h at 37 °C in a GasPak EZ Campy microaerophilic environment container (BD Diagnostics, Sparks, MD). Bacterial colonies with morphology consistent with members of the genus *Listeria* were sub-cultured onto TSA with 5% sheep’s blood for purity.

Sub-cultured, suspect *Listeria* colonies were subjected to gram staining, catalase testing, and were phenotypically tested using a commercial identification platform using manufacturer’s instructions for Protocol A (Biolog, Omnilog, Hayward, CA). Samples that did not have growth consistent with *L. monocytogenes* or *L. ivanovii* on primary isolation media were subjected to PCR testing specific for Listeria hemolysin (*hly*). Nucleic acid was extracted from culture samples in Fraser’s media using a commercial DNA extraction kit (Qiagen, DNA mini kit and QIACube) per manufacturer’s instructions for bacteria. Samples that were negative following PCR testing were placed into cold enrichment (4 °C) for 6 weeks. Cultures with a lack of esculin hydrolysis in Fraser media after 6 weeks of cold enrichment were considered negative for *L. monocytogenes*. No additional diagnostics were performed on *L. monocytogenes* negative submissions.

### *L. monocytogenes* detection and isolation from feed and water

Feed and water samples were screened for the presence of *Listeria* spp. and *L. monocytogenes* using BAX System Real-Time PCR Assays (Dupont Qualicon, Wilmington, DE) and Atlas Detection assays (Roka Bioscience, Lake Forest Park, WA). Water samples were screened directly and after culture enrichment, while feed samples were only screened after enrichment. For direct screening, 1 mL of each water sample was placed in an Atlas G2 Sample Tube (Roka Bioscience) and subjected to testing. Feed samples were enriched for rapid screening by mixing 50 g feed into 200 mL of *Listeria* enrichment broth (LEB; Dupont Qualicon, Wilmington, DE) and water samples were enriched by diluting 10 mL of each water sample into 90 mL of LEB. Samples were incubated at 30 °C for 24 h. After incubation, a 1 mL portion was placed into an Atlas G2 Sample Tube (Roka Bioscience) and a 20 μL portion was used to prepare a BAX template lysis. All Atlas G2 sample tubes were processed through the RokaBioscience Atlas instrument using the Atlas Listeria LSP Assay and the Atlas LmG2 Assay for *Listeria* spp. and *L. monocytogenes* respectively. The BAX lysis preparations from each sample were processed through the BAX Q7 instrument using a BAX System Real-Time PCR Assay for *L. monocytogenes* according to the manufacturer’s instructions.

To isolate and confirm *L. monocytogenes* from the feed and water samples the 24 h LEB enrichments were streaked for isolation onto a Difco Oxford agar plate (Beckton Dickinson and Co., Franklin Lakes, NJ) and a CHROMagar *Listeria* plate (DRG International, Inc., Springfield, NJ) using a sterile cotton swab and inoculating loop. Plates were incubated at 37 °C overnight then viewed for suspect colony phenotypes; black colonies on Oxford agar for *Listeria* spp. and blue colonies without and with halos on chromogenic agar for *Listeria* spp. and *L. monocytogenes* respectively, were targeted. Suspect colonies were selected and placed into a 96-well block containing 1 mL per well of Fraser media containing 5% ferric ammonium citrate, and then incubated at 37 °C overnight.

The 24 h LEB enrichments of feed and water were further incubated for another 24 h (48 h total) and the above streaking for isolation onto chromogenic *Listeria* and Oxford agar was repeated. A secondary 48 h enrichment in Fraser media was incubated an additional 48 h at 30 °C and was streaked for isolation, incubated and viewed for suspect colonies as described above.

Suspect colonies were confirmed to the species level using a *Listeria* spp. specific PCR and biochemical tests. *Listeria* species *monocytogenes, innocua, grayi, ivanovii, seeligeri* and *welshimeri* were identified through *Listeria* spp. multiplex PCR [36]. The isolates that were identified as *L. monocytogenes* were further characterized using the serovar multiplex PCR [21]. Suspect isolates that were found to possess the phosphoribosyl pyrophosphate synthetase (*prs*) gene, indicative of all *Listeria* spp., but which could not be identified through PCR were further identified using biochemical tests. Each suspect *Listeria* was streaked for isolation on tryptic soy agar containing 0.6% yeast extract, incubated at 37 °C overnight and then processed using a Remel Micro-ID Listeria Kit (Thermo Fisher Scientific, Lenexa, KS) according to the manufacturer’s protocol.

### *L. monocytogenes* strains selected for sequencing

A total of 31 *L. monocytogenes* strains were selected for whole-genome DNA sequencing on a MiSeq instrument (Illumina, San Diego, CA). Of those, 19 clinical strains were isolated from USMARC cattle aborted fetuses, placentas, or uterine swabs. Another eight strains were isolated from four different corn silage samples, three water tanks and one standing water source. Additionally, four other strains from bovine abortions were obtained from the UNLVDC that originated from other regions in Nebraska for use as controls and references.

### DNA preparation and Illumina MiSeq whole-genome sequencing

All 31 *L. monocytogenes* strains were passaged twice from − 80 °C frozen stocks on chocolate agar plates (Hardy Diagnostics, Santa Maria, CA) at 37 °C. A single colony of each isolate was then inoculated in 1.5 mL of Brain-Heart Infusion (BHI) broth and grown overnight without shaking at 37 °C. Genomic DNA was extracted from the cultures using MO BIO microbial DNA isolation kits (MO BIO Laboratories, Carlsbad, CA) according to the manufacturer’s instructions. The extracted DNAs were quantified and checked for purity using 260/280 absorbance readings on a NanoDrop ND-1000 spectrophotometer (NanoDrop, Wilmington, DE). Individual libraries were constructed for each of the strain DNA preparations using Illumina Nextera XT DNA sample preparation kits with appropriate indices tags according to the manufacturer’s instructions (Illumina Inc., San Diego, CA). The libraries were pooled together and run on an Illumina MiSeq DNA sequencer (Illumina Inc., San Diego, CA). The genome of each strain was sequenced to a minimal depth of 10X coverage.

### Assembly of *L. monocytogenes* chromosomes and phylogenetic trees

Adapter sequence and low-quality bases were trimmed using Trimmomatic [37]. Trimmed fastq reads were assembled using SKESA. The *L. monocytogenes* assembled chromosomes from 27 unique USMARC strains and 4 clinical strains from the UNLVDC were imported into parsnp [38] for genome alignments and subsequent identification of core-genome SNPs and construction of a preliminary phylogenetic tree. The strains grouped into six initial clusters. One strain from each cluster was selected for PacBio sequencing to obtain a complete closed chromosome representative of each cluster (described below). Illumina reads from strains not also sequenced with PacBio were assembled using SKESA. The SKESA, PacBio, and 20 closed *L. monocytogenes* chromosomes from GenBank [39] were then used in parsnp to create a new phylogenetic tree. The 20 GenBank chromosomes represented all four known *L. monocytogenes* lineages and were utilized to calibrate the final phylogenetic tree. Evolview was used to populate the tree with phenotypic metadata [40].

### DNA preparation and PacBio whole-genome sequencing library construction

High molecular weight DNA was extracted from *L. monocytogenes* cultures using Qiagen Genomic-tip 100/G columns and a modified manufacturer’s protocol as previously described [41] with the addition of mutanolysin with the proteinase K step followed by incubation at 50 °C for 1 h. Ten micrograms of DNA were sheared to a targeted size of 20 kb using a g-TUBE (Corvaris, Woburn, MA) and concentrated using 0.45X volume of AMPure PB magnetic beads (Pacific Biosciences, Menlo Park, CA) following the manufacturer’s protocol. Sequencing libraries were created using 5 μg of sheared, concentrated DNA and the PacBio DNA Template Prep Kit 2.0 (3Kb - 10Kb) according to the manufacturer’s protocol. The library was bound with polymerase P5 followed by sequencing on a Pacific BioSciences (PacBio) RS II sequencing platform with chemistry C3 and the 120 min data collection protocol.

### PacBio sequence assembly into closed circularized genomes

PacBio reads were assembled using HGAP3 (SMRTanalysis Version 2.1) and the resulting contigs were imported into Geneious. Within Geneious, overlapping sequence on the ends of the contigs were removed from the 5′ and 3′ ends to generate a circularized chromosome. The chromosome was reoriented to start with a putative origin of replication with Ori-Finder [42]. The chromosome was initially polished for accuracy using the Resequencing 1.0 protocol in SMRTanalysis by mapping corrected PacBio reads to the chromosome. To correct PacBio sequencing errors (homopolymers and SNPs), Illumina reads were mapped to the initially polished chromosome using Pilon. Then, both PacBio and Illumina reads were mapped to the Pilon-generated chromosome using Geneious Mapper. Additional sequencing errors were identified and corrected by manual editing in Geneious, resulting in a finished closed circularized chromosome. Chromosome sequences were deposited into NCBI (Additional file 2) and annotated using the Prokaryotic Genome Annotation Pipeline version 4.5. MLST 2.0 [43] was used to determine the MLST allelic profiles for the *L. monocytogenes* strains from USMARC and UNLVDC. MLST allelic profiles were then used with the Listeria Pasteur MLST database to determine serotype, lineage and clonal complex [21, 22].

## Supplementary information

**Additional file 1.** Sampling scheme of corn silage and earlage piles. Image of silage face with grid overlay. Eight samples (ovals; 1–8) were collected from each pile according the gridded lines. Loose surface materials (9–10) and the drainage pipe (11) were also collected.

**Additional file 2 ***L. monocytogenes* strains used in the project with corresponding genotypes, sequencing information and sample type.

**Additional file 3 **Gingr visualization of the genomes of lineage III *L. monocytogenes* strains from cattle, water or feed associated with the outbreak. The genomes were aligned with parsnp. The 19 strains in this figure comprised the monophyletic clade in Fig. 2. The outer taxonomic units in the phylogenetic tree are aligned with their corresponding row in the alignment. Each row represents the entire genome for a strain. Vertical purple lines in the alignment indicate base differences. Asterisks are to the right of the six strains whose core genomes are identical with their names highlighted according to sample type; cattle (green), water (blue) or feed (red). (PPTX 75 kb)

## Data Availability

The Illumina sequencing reads for the 31 isolates sequenced on that platform have all been placed in the NCBI sequence read archive (SRA) for public availability. Additionally, the complete genomes of all six isolates sequenced and assembled with PacBio and Illumina sequences are publicly available in GenBank (CP032668-CP-32673).

## Abbreviations

CV: Cervico-vaginal
MLST: Multi-locus sequence typing
NAHMS: National Animal Health Monitoring System
NCBI: National Center for Biotechnology Information
PFGE: Pulse-field gel electrophoresis
RFM: Retained fetal membranes
SKESA: Strategic Kmer Extension for Scrupulous Assemblies
SNPs: Single nucleotide polymorphisms
UNLVDC: University of Nebraska, Lincoln, Veterinary Diagnostic Center
USMARC: United States Meat Animal Research Center
