# Supplementary material for: Genomic-based identification of environmental and clinical Listeria monocytogenes strains associated with an abortion outbreak in beef heifers
Source: BMC Vet Res. 2020 Feb 22;16:70. doi: 10.1186/s12917-020-2276-z (PMC7036198; doi:10.1186/s12917-020-2276-z)
Supplement: Supplementary file 1 — Additional file 1. Sampling scheme of corn silage and earlage piles. Image of silage face with grid overlay. Eight samples (ovals; 1–8) were collected from each pile according the gridded lines. Loose surface materials (9–10) and the drainage pipe (11) were also collected. [file 12917_2020_2276_MOESM1_ESM.docx]

**Figure S1**: Sampling scheme of silage and earlage piles. Eight samples (ovals; 1-8) were collected from each pile according the gridded lines. Loose surface materials (9-10) and the drainage pipe (11) were also collected.


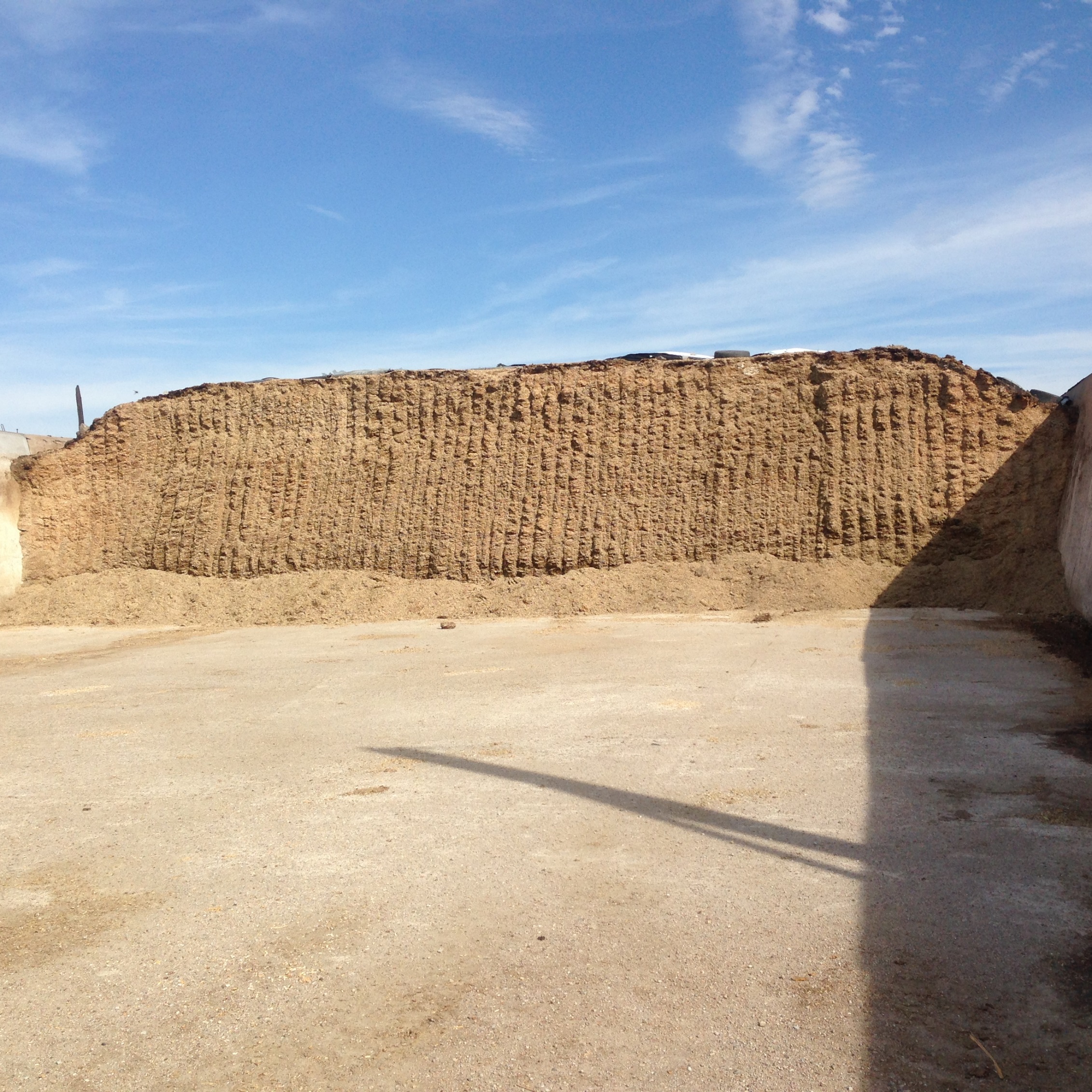
*
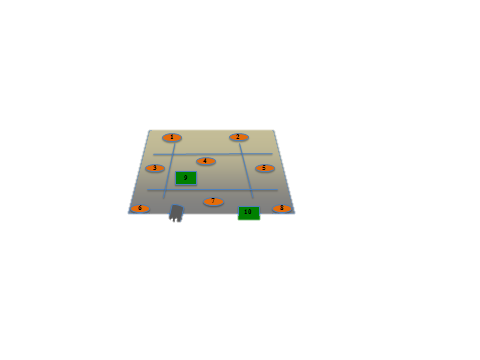
*

**11**
