# Supplementary material for: Genomic-based identification of environmental and clinical Listeria monocytogenes strains associated with an abortion outbreak in beef heifers
Source: BMC Vet Res. 2020 Feb 22;16:70. doi: 10.1186/s12917-020-2276-z (PMC7036198; doi:10.1186/s12917-020-2276-z)
Supplement: Supplementary file 3 — Additional file 3 Gingr visualization of the genomes of lineage III L. monocytogenes strains from cattle, water or feed associated with the outbreak. The genomes were aligned with parsnp. The 19 strains in this figure comprised the monophyletic clade in Fig. 2. The outer taxonomic units in the phylogenetic tree are aligned with their corresponding row in the alignment. Each row represents the entire genome for a strain. Vertical purple lines in the alignment indicate base differences. Asterisks are to the right of the six strains whose core genomes are identical with their names highlighted according to sample type; cattle (green), water (blue) or feed (red). (PPTX 75 kb) [file 12917_2020_2276_MOESM3_ESM.pptx]

## Slide 1
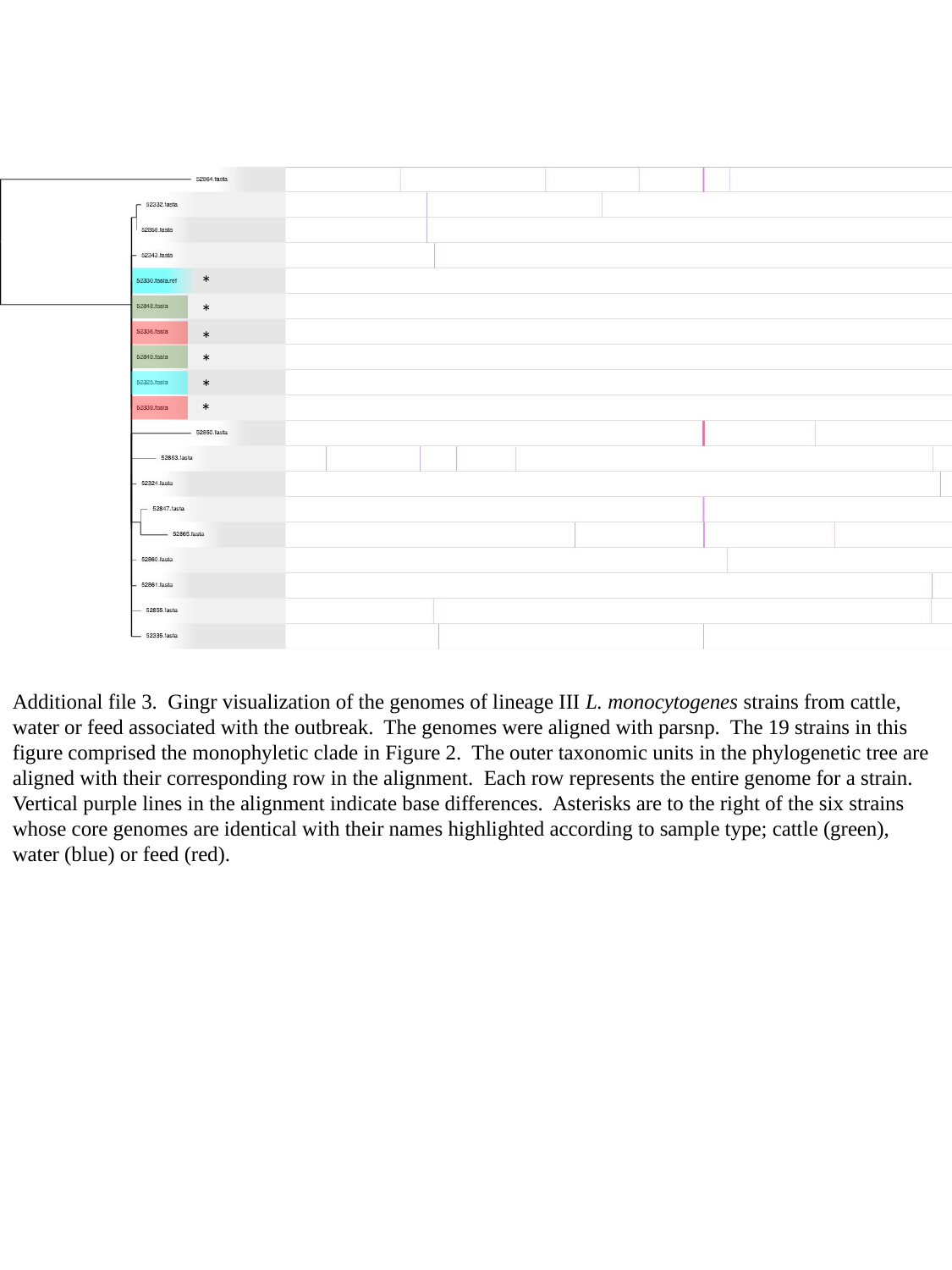

*
*
*
*
*
*
Additional file 3. Gingr visualization of the genomes of lineage III L. monocytogenes strains from cattle, water or feed associated with the outbreak. The genomes were aligned with parsnp. The 19 strains in this figure comprised the monophyletic clade in Figure 2. The outer taxonomic units in the phylogenetic tree are aligned with their corresponding row in the alignment. Each row represents the entire genome for a strain. Vertical purple lines in the alignment indicate base differences. Asterisks are to the right of the six strains whose core genomes are identical with their names highlighted according to sample type; cattle (green), water (blue) or feed (red).
